# A passion for aging in cultural contexts: Dr. Colette Browning and her contributions within Australia and globally

**DOI:** 10.3389/fpubh.2022.1017368

**Published:** 2022-12-05

**Authors:** Nancy A. Pachana

**Affiliations:** School of Psychology, Health and Behavioral Sciences Faculty, The University of Queensland, St. Lucia, QLD, Australia

**Keywords:** aging, culture and health, longitudinal studies, public health policy, mentoring, social determinants of health, chronic disease management

We are increasing a world characterized by movement, particularly the movement of people. I am part of that movement, a clinical geropsychologist trained in the United States, who has migrated first to New Zealand and who now finds her home in Australia. However, cultural contexts, whether they be original or adopted, have always intrigued me and informed my research and clinical practice with older adults. Thus, it is no surprise that upon arrival in Australia I found a kindred spirit in Colette Browning who personifies excellence in aging research in Australia and globally.

Professor Colette Browning is a health psychologist and social gerontologist with a BSc (Hons) and MSc in psychology and a PhD in public health, currently working at Federation University in Australia. She is an international leader in health psychology, with a research focus on public health and aging, particularly in diverse cultural contexts. Her interests led her to have strong collaborations with colleagues in China, where her research focused primarily on health care reform, aging and health services evaluation in that country. We often had long discussions about the insights each of us has gathered working with overseas colleagues, comparing our international research experiences and marveling at the diversity of policy and practice variation cross-nationally.

Closer to home in Australia, Colette's research focuses on healthy aging, services for older people, quality of life for older people, and behavioral and social factors in aging and chronic disease self-management. For many years she co-directed the Melbourne Longitudinal Studies on Healthy Aging program (MELSHA) with Professor Hal Kendig, himself a leading light in Australian aging research. The MELSHA 20-year longitudinal study on aging established an evidence base to inform health promotion programs for older people, a relatively novel idea when it was first established in the 1990's, as most research on aging in Australia at that time focused on the “burden of aging” and had not really embraced the personal and societal gains of increased longevity. The distance traveled in that time is evident in the UN declaration of the Decade of Healthy Aging, where we are now.

I consider Colette both a colleague and a mentor, invaluable when one is new to a place / culture / society. I joined the Australian Psychological Society very soon after arriving in Australia in 2000, and found a host of colleagues in the gerontological space, including Colette. Together we have planned symposia and supported both new and more established colleagues in their research endeavors, including getting research implemented in practice. In recognition of her many contributions to the psychology profession over time, she is a Fellow of the Australian Psychological Society (APS) as well as the APS College of Health Psychology, and is much valued by younger colleagues for her mentoring skills.

The movement of people often takes them across institutions, and Colette has held a variety of roles at several of Australia's leading universities, as well as in overseas institutions. Colette holds Honorary professorial positions at Peking University, China and the Research School of Population Health at the Australian National University in Canberra. For a time, she was the Research Director of the International Primary Health Care Research Institute, in Shenzhen, China. She was also previously the Director of the Royal District Nursing Service (RDNS) Institute, an opportunity for her to implement age-related research into practice on the ground. She has also been Director of Research and Professor of Healthy Aging in the School of Primary Health Care at Monash University. Of course, having held so many positions directing research at a high level, it was inevitable that she would rise to the level of Associate Dean Research at yet another well-regarded university for aging research, namely La Trobe University. This wealth of institutional and management experiences was personally invaluable to me, as I progressed from a largely clinical practice and research background through more managerial and higher level research positions over the course of my own career. Colette's advise on navigating the complexities of accreditation guidelines and policy (part of an important role I undertook at a national level for 5 years) was one example of her mentoring, where her experience with stakeholders with very different agendas, drivers and worldviews was invaluable to me in my development as a leader and influencer of policy in my own right.

Extensive experience in teaching and curriculum development in the areas of gerontology, research methods and psychosocial aspects of health led to her management of the Australian Corporate Public Health Postgraduate Program. This program is specifically designed for staff in the Commonwealth Department of Health and Aged Care, with a focus on policy issues in public health. As a foundational member of the national consortium that developed this national postgraduate curriculum on aging under the Public Health Education and Research program, Colette's work has touched the lives of many aspiring scholars and practitioners.

Colette has a career total of over 260 publications including well-regarded peer-reviewed papers, book chapters, and authored books making her one of the most prolific researchers in aging and public health. Her published work spans an amazing array of topics, across both primary research and policy foci. Some of her most cited work has focused on sensory impairments (1, 2), falls (3), and illness and disability (4) in later life. Implementation of research into practice is an important part of her legacy, as is cross-disciplinary research (5, 6). Her research expertise includes longitudinal and mixed methods approaches, systematic reviews, program evaluations, and randomized controlled trials. Her research on the social determinants of health (7) foreshadowed the burgeoning body of work in this area. An exciting aspect of her new work at the Health Innovation and Transformation Center at Federation University over the next 5 years, is a program of research on chronic disease prevention and management at the intersection of behavioral, genomics, and digital health interventions building on her work in Australia and China (8).

Throughout her career, Colette has taken a keen interest in how cultural aspects of older adults must inform their access to care. Whether in access to dementia services (9), management of chronic health conditions (10), or training in diversity for health and aged care workers (11), Colette's contributions in the cross-cultural space will be her most lasting contributions to women in science. I personally am extremely grateful for her friendship, collegiality, and mentoring of a transplanted American in Australia.

## Author contributions

The author confirms being the sole contributor of this work and has approved it for publication.

## Conflict of interest

The author declares that the research was conducted in the absence of any commercial or financial relationships that could be construed as a potential conflict of interest.

## Publisher's note

All claims expressed in this article are solely those of the authors and do not necessarily represent those of their affiliated organizations, or those of the publisher, the editors and the reviewers. Any product that may be evaluated in this article, or claim that may be made by its manufacturer, is not guaranteed or endorsed by the publisher.
